# 

**Published:** 1895-06-01

**Authors:** 


**Notices of Births, Deaths, and Marriages** are inserted in this column at a charge of 2s. 6d. for an announcement not exceeding four lines.

**Notice to Advertisers and Subscribers.**

All Advertisements, Orders for Copies of The Hospital and Business Communications should be addressed to **The Manager, NOT TO THE EDITOR,** The Hospital, 428, Strand, London, W.C.

The Cost of Subscription, post free, is as follows:—Per week, 2½d.; per quarter, 2s. 8d.; per half-year, 5s. 3d.; per annum, 10s. 6d. Foreign:—Per Annum, 15s. 2d.; payable, in all cases, in advance.

**NOTICE TO CORRESPONDENTS.**

All MS., Letters, Books for Review, and other matters intended for the Editor should be addressed The Editor, The Lodge, Porchester Square, London, W.

The Editor cannot undertake to return rejected MS., even when accompanied by stamped directed envelope.

**"Burdett's Hospital Annual," 1895,**

Ready Immediately.

Sixth year of publication. About 1000 pp. Scarlet cloth, gilt lettered. Price 5s. A most complete guide to British, Colonial, Indian, and American Hospitals, Dispensaries, Nursing and Convalescent Institutions, and Asylums. A few copies of the "Annual" for 1894 may still be obtained on application to the Publishers, The Scientific Press (Limited), 428, Strand, London, W.C.

**NOTICE.—Vol. XVII. of "The Hospital" (October, 1894, to April, 1895), in handsome cloth binding, is now on sale at the Office of this Journal. Price 6s. Cases for binding the Numbers contained in this Volume, 1s. 6d. Index to Vol. XVII., 2½d., post free.**

### SCRAPS AND GLEANINGS.

A bacteriological chair has been created at the Medical College at Nantes.

At the North Brierly Joint Hospital Board, 107 patients have been admitted during the year.

The degree of Ph.D. has been conferred upon Miss Grace Chisholm of the University of Göttingen.

Our French contemporary, the *Progrès Médical*, gives the number of the blind in France at about 40,000.

The memorial stone of the New Samaritan Hospital, at Glasgow, was laid last week with Masonic honours.

Her Majesty has forwarded, through Sir Fleetwood Wilson, a donation of £50 to the Royal Maternity Hospital.

At Louvain, in Belgium, his birthplace, a monument has been erected to Father Damien, the good apostle to the lepers.

The French Institute attains its centenary in the coming autumn. Fêtes, &c., are being arranged in order to celebrate the event.

In Chicago one firm of lawyers exist solely for the business of collecting doctors' bills, and the business they do is an extensive one.

An appeal is on foot for further support for the Newbury District Hospital, the committee of which are under some anxiety as to its finances.

Four philanthropic women in Buffalo have contributed a sum of 55,000 dollars towards the building fund of the new Local General Hospital.

The Lord-Lieutenant of Ireland last week opened a bazaar in the Royal Dublin Society's grounds at Ballsbridge, in aid of the funds of Sir Patrick Dun's Hospital.

The annual report of the Meath Hospital and County Dublin Infirmary is a creditable one on all points, whilst the financial year has opened with a credit balance of £269.

In addition to the £206 received and promised for the building fund of the Royal Surrey County Hospital, a cheque for £50 has been received from Mr. C. McAndrew, of Bramley.

Messrs. Studt and Sons gave a brilliant and successful fête at Leominster last week in aid of the local cottage hospital, and £62 was handed over to the authorities as the outcome of the same.

Electric heat has been applied with success to the thawing out of frozen water pipes in England. A wire is run into the pipe until it meets the obstruction and then the current is turned on.

At the annual festival dinner of the Provident Surgical Appliance Society subscriptions and donations amounting to £1,000 were received, including £105 from the chairman, Mr. M. P. Grace.

The amount subscribed by the public works towards the expenses of the Glasgow Ear Hospital, shows that the work of the hospital is appreciated, by those for whom it is primarily intended.

Sir Joseph Lister was awarded the Albert Medal of the Society of Arts, for the antiseptic methods he has applied to surgery. The medal was personally presented by H.R.H. the Prince of Wales.

The fiftieth anniversary of the formation of the Dudley Dispensary has now been reached, and a special meeting of subscribers been called for the purpose of formulating a scheme to celebrate the event.

The oldest man in the world lives in Russia, he is 126 years of age. An account of his life has just appeared in print. By birth a Frenchman, he was one of the 400,000 who followed Napoleon to Moscow.

The formal opening of the new wing which has been added to the West of Scotland Convalescent Seaside Homes at Dunoon for the accommodation of Women and Children took place on Saturday in last week.

Count Tolstoi has become a devoted cyclist, and a member of the Moscow Cycling Club. Report says that he rides for an hour each day, accompanied by various members of his family, and that he has mastered the art in a very short time.

The Health and Sanitary Committee of Haslingden have resolved that it is desirable a smallpox hospital should be erected for the locality, and have appointed a sub-committee to report as to the most suitable plan of hospital to erect, as also on the choice of site.

A two days' sale of work was held last month in the interest of the Sunderland Eye Infirmary, the object of the sale being to raise funds to assist the out-patients of the institution to obtain rest, nourishment, and spectacles, &c., as ordered by the medical staff.

Mr. Victor Horsley presided at the last meeting of the Society for the Prevention of Hydrophobia and the Reform of Dog Laws. This society has as its object the total extinction of rabies by a system of universal muzzling for a definite period, and further through a strict and permanent quarantine.

The late Lord Drumlanrig was deeply interested in the Society for the Prevention of Cruelty to Children, and it was therefore a happy thought which dictated that the sum of £2,000, subscribed as a tribute to his memory, should be paid over to the credit of the society whose welfare he had so much at heart.

The annual report of the North Eastern Hospital for Children shows the number of out-patients treated as 13,124 during the period under review. An attempt is being made to revive the Children's Association, founded in connexion with the hospital in 1877, and a committee of ladies have been appointed to carry out the same.

Nearly 500 children have been sent from the Victoria Hospital for Children, Chelsea, to the Convalescent Home at Broadstairs. Contributions to the extent of £1,200 have been received in support of the "Infant Prince Fund," a fund which was started at the Hospital to commemorate the birth of a Prince to the throne.

At the annual meeting of the Newcastle Eye Infirmary just held, a falling off of the income was noted. In order to discharge existing liabilities, and to provide for current expenses, the committee have been obliged to realise a portion of the invested property, and inroads into the accumulated capital will soon have to be made unless further subscriptions are forthcoming.

### The Student of Medicine.

### Appointments.

T. H. Agnew, L.R.C.S., L.R.C.P.Edin., appointed House Physician to the Liverpool Royal Infirmary. H. Armstrong, M.B., Ch.B.Vict., appointed House Surgeon to the Liverpool Royal Infirmary. R. E. Bickerton, M.B., Ch.B.Vict., appointed House Physician and Ophthalmic Assistant to the Liverpool Royal Infirmary. R. D. Bradford, L.R.C.P., L.R.C.S.Edin., appointed Medical Officer for the Adbro' District of the Skirlaugh Union. Mr. T. Bridewell, appointed Medical Officer for the Chigwell District of the Epping Union. Mr. T. Bundle, appointed Medical Officer for the Southwick District of the Fareham Union. A. Campbell-Smith, M.B., C.M.Edin., appointed Dispensary Surgeon to the Bradford Infirmary. T. Carwardine, M.B., M.S.Lond., F.R.C.S.Eng., appointed House Surgeon and Senior Resident Officer to the Bristol Royal Infirmary. Mr. A. H. Collins, appointed Medical Officer for the Riseley District of the Bedford Union. F. A. Elkins, M.B., C.M.Edin., appointed Medical Superintendent to the Sunderland Borough Asylum. J. E. Finnie, M.R.C.S., L.R.C.P., appointed House Surgeon to the Lock Hospital, Liverpool. T. J. Fletcher, M.B., C.M.Edin., M.R.C.S., appointed Medical Officer and Public Vaccinator for the Castle Donington District of the Shardlow Union. F. W. A. Godfrey, M.B., C.M.Edin., appointed Honorary Surgeon to the Scarborough Hospital and Dispensary. Mr. T. Green, appointed Medical Officer for the Second District of the Cambridge Union. A. C. Hartley, M.D.Edin., F.R.C.S.Edin., appointed Medical Officer for the Bedford and Kempston District of the Bedford Union. A. Howie, M.B., C.M.Glasg., appointed Medical Officer

THE STUDENT OF MEDICINE—*continued.*

for the Westbury District of the Atcham Union. W. Hunter, M.D., M.R.C.P., appointed Pathologist and Curator of Museum to the Charing Cross Hospital. J. L. Johnstone, M.R.C.S.Eng., L.R.C.P.Lond., appointed Medical Officer and Public Vaccinator for the Upholland and Dalton Districts of the Wigan Union. J. A. Malcolmson, M.D.Q.U.I., L.M., reappointed Medical Officer of Health to the Eston Urban District Council. R. Odell, L.R.C.P.Lond.,M.R.C.S.Eng.,appointed Medical Officer forThird District of the Hertford Union. A. M. Palmer, L.R.C.P.Edin., M.R.C.S.Eng., reappointed Medical Officer of Health to the Whittington Urban District Council. S. J. Ross, M.B., Ch.B.Vic., appointed House Surgeon to the Liverpool Royal Infirmary. G. T. Schofield, L.R.C.P., L.R.C.S.Edin., appointed Medical Officer and Public Vaccinator for the Twelfth (Mossley) District of the Ashton-under-Lyne Union. E. Smallwood, L.S.A., appointed House Physician to the Liverpool Royal Infirmary. E. H. W. Swete, M.D.St.And., M.R.C.S., reappointed Medical Officer of Health to the Droitwich Rural District Council. Leo. Taylor, F.I.C., F.C.S., appointed Public Analyst for Hackney, vice Dr. Tripe, deceased. J. Kay Tomory, M.B., C.M.Edin., appointed Public Vaccinator for the Parish of Halkirk. Dr. Walker, reappointed Medical Officer of Health to the Wrotham Urban District Council. A. Meredith Whitehead, M.B., C.M.Aberd., M.R.C.S.Eng., appointed Honorary Surgeon to the Wellington Hospital, New Zealand. J. Howard Wilkinson, M.R.C.S.Eng., L.R.C.P.Lond., appointed Honorary Surgeon to the Dudley Dispensary. H. R. Wilson, M.R.C.S., L.R.C.P., appointed House Surgeon to the Liverpool Royal Infirmary.

### VACANCIES.

The following vacancies are announced this week, the amount of salary in each case being given after the name of the institution:

### Resident Medical Officers.

City of London Hospital for Diseases of the Chest, Victoria Park, E.—House Physician. Board, residence, and allowance for washing provided. Appointment for six months. Applications to the Secretary, at the office, 24, Finsbury Circus, E.C., by June 13th.

Durham County Asylum.—Pathologist and Junior Resident Medical Officer. Salary £100 per annum. Applications to the Superintendent, Durham County Asylum, Winterton, Ferryhill, by June 8th.

General Hospital, Birmingham.—Assistant House Surgeon. Must possess surgical qualification and be registered. Appointment for six months. No salary, but residence, board, and washing provided. Applications, with certificate of registration and copies of testimonials, to H. J. Collins, House Governor, by June 1st.

Hospital for Paralysed and Epileptic (Albany Memorial), Queen Square, Bloomsbury.—Senior and Junior House Physician. Salary for the former £100, and for the latter £50 per annum. Board and apartments in the hospital provided in each case. Application to B. Burford Rawlings, Secretary and General Director, by June 4th.

Hospital for Sick Children, Newcastle-on-Tyne.—Resident Medical Officer; doubly qualified. Salary £60, with board, lodging, and laundry. Applications to the Secretary by June 6th.

Liverpool Stanley Hospital.—Junior House Surgeon. Must possess a registered medical and surgical qualified qualification. One who has filled a similar office preferred. Salary £70 per annum, with board, &c. Applications and testimonials by May 30th. Also Honorary Assistant Surgeon. Applications to J. E. Bennett, Honorary Secretary, by June 5th.

North-Eastern Hospital for Children, Hackney Road, Shoreditch, N.E.—House Physician. Appointment for six months, and after the expiration of this term the House Physician will be required, if eligible, to serve as House Surgeon for a further period of six months. Must possess a medical and surgical qualification. Salary as House Physician at the rate of £60 per annum, and as House Surgeon at the rate of £80 per annum. Applications and testimonials to T. Glenton-Kerr, Secretary, City Office, 27, Clement's Lane, Lombard Street, E.C., by June 11th.

Northern Infirmary, Inverness.—House Surgeon and Dispenser. Salary £70 per annum, with board, &c. Applications to Mr. Duncan Shaw, Honorary Secretary, by June 5th.

Royal Ear Hospital, Frith Street, Soho, W.—House Surgeon. Appointment for six months. Honorarium £12 12s. Application to D. Murray, Honorary Secretary, by June 8th.

Royal Free Hospital, Gray's Inn Road, W.C.—Two Resident Medical Officers. Doubly qualified. Appointment for six months, but the holders will be eligible for re-election. No salary, but board, residence, and washing. Applications and testimonials to Conrad W. Thies, Secretary, by June 1st.

Trinity College, Dublin.—Lecturer in Pathology. Applications to the Registrar of the University by June 15th.

West London Hospital, Hammersmith Road, W.—House Physician and House Surgeon. Appointments for six months. Board and lodgings provided. Applications to R. J. Gilbert, Secretary-Superintendent, by June 19th.

### Non-Resident Medical Officers.

Dental Hospital of London, Leicester Square.—Two Dental Surgeons. Candidates must be Licentiates of Dental Surgery of one of the licensing bodies recognised by the General Medical Council. Applications and testimonials to J. Francis Pink, Secretary, by June 10th.

London County Council.—Pathologist to the London County Asylums. Salary £700 per annum, and travelling expenses. Applications (on forms provided), endorsed "Applications for Pathologist," to R. W. Partridge, Clerk to the Asylums Committee, 21, Whitehall Place, S.W., by June 17th.

Royal College of Surgeons of England.—Hunterian Professors, the Erasmus Wilson Lecturer, and the Arris and Gale Lecturer for the ensuing year. Applications, with particulars of subjects upon which it is proposed to lecture, to E. Trimmer, Secretary, by June 6th. Also two Examiners in Dental Surgery; applications by same date.

Royal Westminster Ophthalmic Hospital, King William Street, West Strand.—Clinical Assistants. Appointments for six months. Must be duly qualified, and preference will be given to those who have had previous experience of ophthalmic practice. Applications and testimonials to T. Beattie-Campbell, Secretary, by June 1st.

**Medical Appointment.**

NORTH-EASTERN HOSPITAL for CHILDREN, HACKNEY ROAD, SHOREDITCH, N.E.

T. GLENTON-KERR, Secretary.

